# Goal Setting for Participatory Person-Centered Geriatric Rehabilitation—From Function-Centered Rehabilitation towards Digitally Supported Personalized and Integrated Care for Older People

**DOI:** 10.3390/jcm13144134

**Published:** 2024-07-15

**Authors:** Martin Skoumal, Sonja Lindner-Rabl, Martina Honegger, Christoph Pertinatsch, Christof Kadane, Britta Neubacher, Carolin Herzog, Regina Roller-Wirnsberger

**Affiliations:** 1 Department of Internal Medicine, Research Unit for Aging and Life Long Health, Medical University of Graz, 8036 Graz, Austria; martin.skoumal@medunigraz.at (M.S.); martina.honegger@medunigraz.at (M.H.); christoph.pertinatsch@medunigraz.at (C.P.); christof.kadane@medunigraz.at (C.K.); britta.neubacher@medunigraz.at (B.N.); carolin.herzog@medunigraz.at (C.H.); 2 Department for Scientific Research in Rehabilitation, Pension Insurance Austria, 1021 Vienna, Austria

**Keywords:** geriatric rehabilitation, goal setting, ICF, prevention, digitalization

## Abstract

As chronic illness is common among older people, self-care practices for older people are needed to control health status, to prevent possible complications and to ensure optimal quality of life. The literature has demonstrated that integrated care approaches are one key success factor for delivering person-centered and sustainable care for older people, with rehabilitation being a cornerstone in tertiary care prevention for older citizens. The current paper addresses the state of the literature for person-centered geriatric rehabilitation (GR) and the importance of personalized and participatory goal setting. In accordance with the bio–psycho–social model of the International Classification of Functioning, Disability and Health (ICF), social participation and the related goals are of particular importance for the entire rehabilitation process. The social participation of individuals enrolled into GR is therefore one of the milestones to be achieved during GR. Personalized goal setting during the entire rehabilitation process, Comprehensive Geriatric Assessment (CGA) and shared decision making allow a comprehensive care approach separate from solely function-based rehabilitation. The review also focusses on recent developments in digitalization in healthcare and delivers insights into how healthcare professionals’ collaborative practice supports sustainable rehabilitation results in patients of advanced chronological age.

## 1. Introduction

By 2050, 129.8 million people—almost 30% of the European population—will be 65 years or older [1,2]. Alongside physiological ageing and multimorbidity, the prevalence of reduced functional capacity and/or frailty increase with chronological age. The prevalence of frailty and reduced functional capacity in people older than 65 years and living at home is approximately 10% across Europe [3]. Within the different frailty models, reduced participation and consequent care dependence are crosslinking elements as outcome indicators of the trajectories of frailty [4,5]. Therefore, not only recognition of the physical functional capacities but also of the social and behavioral factors associated with frailty may influence rehabilitation interventions for individuals at risk of worsening frailty, specifically when targeted at younger “old age” [6].

Within the concept of “integrated care for older and/or frail people” [7,8,9,10], geriatric rehabilitation (GR) has become a cornerstone of care for patients affected by multimorbidity and geriatric syndromes who have the potential to improve their functional performance and participation outcomes [11]. In many European countries, the focus of GR programs is on functional assessments and changes in functional outcome parameters [12,13]. This approach is rooted in the uniform use of the International Classification of Functioning, Disability and Health (ICF) framework, published by the World Health Organization (WHO) [14] in rehabilitation medicine across many European countries. The ICF clusters functional domains into ICF core sets, allowing an individualized description of timely evaluated functional capacities on patient level as well as the monitoring of functional capacity and health alongside the rehabilitation process [8]. This approach is reflected in many rehabilitation programs across Europe. Many of the rehabilitation programs offered to older people have a strong focus on restoring physical functional capacity as the major success and outcome parameters. However, within the bio–psycho–social background of ICF, health is defined as the interaction between body functions and their impairments, activities and their limitations and participation and its restrictions by also taking into account contextual, environmental and personal factors. Based on this framework, health may be improved by reducing impairments, helping the performance of activities—even in the face of restricted capacity—and promoting participation even when activity is limited [8]. This also refers to the rehabilitation process itself. Therefore, the ICF offers a taxonomy of information able to support and inform person-centered rehabilitation alongside the trajectories of frailty based upon the bio–psycho–social model of care [15,16,17].

A key driver for person-centered rehabilitation outcomes in GR is goal setting between healthcare professionals and clients. Setting goals in rehabilitation has a longstanding tradition, as individual goals are described based on their timing, abstractness and content [18]. These characteristics are independent from care setting; however, the timing and content are often related to the care setting of rehabilitation (inpatient or ambulatory rehabilitation).

The most common setting for GR is inpatient rehabilitation [19]. However, there is increasing evidence that outpatient GR is of clinical value, especially when evaluated from a patient’s perspective [20]. In this sense, offering GR in a community context means that clinicians need scheduled time specifically for goal-setting meetings [21].

In this community context, digitally supported interventions such as telerehabilitation offer new opportunities, including for GR. There is promising evidence for the sustainable efficacy and effectiveness of telerehabilitation for monitoring and guiding the rehabilitation of older people, including those living at home. According to recent data, technology use was safe even for people with little experience in digital media and those living with cognitive impairments/frailty [22]. However, evidence on the sustainability of digital solutions in person-centered goal setting in rehabilitation is a matter of debate. This is also one of the reasons why eHealth solutions are not yet fully integrated into GR in routine clinical care [23].

The review tries to summarize the key indicators that have an impact on the person-centered and participatory rehabilitation outcomes of geriatric patients, independent from their leading disease and acute events driving them into GR. The publication builds upon the current literature and tries to reflect evidence towards structural and procedural standards for modern GR as part of an integrated care model for older citizens.

## 2. Materials and Methods

A review of the existing literature on goal setting within the framework of shared decision making in rehabilitation settings was undertaken. A literature search was performed in the databases Medline (via PubMed), CINAHL (via EBSCOhost), Cochrane (via Ovid) and Embase (via Ovid). The search strategy comprised of the following terms: “shared decision-making” and “goal setting” or “goal planning” or “goal attainment” and “rehabilitation”. In addition to selected keywords, controlled vocabulary terms like Medical Subject Headings (MeSH) were applied where possible and adjusted to particular database settings. Google Scholar was searched for additional grey literature and further references were determined by manual reference tracking. The search was limited to reviews in English or German published between January 2000 and May 2024. Article screening was conducted independently by two authors, with any discrepancies resolved through consultation with a third reviewer.

## 3. Results

### 3.1. Results from the Literature Search

A total of 52 results were retrieved with the implemented search strategy; 72 additional results were acquired on Google Scholar and via manual reference tracking. Following deduplication (*n* = 8 results removed), title/abstract screening (*n* = 22 results removed) and fulltext screening (*n* = 84 results removed), a total of 10 articles was included in this review. The PRISMA 2020 flow diagram delineates the screening process and explicates the reasons for exclusion.

**Figure 1:**
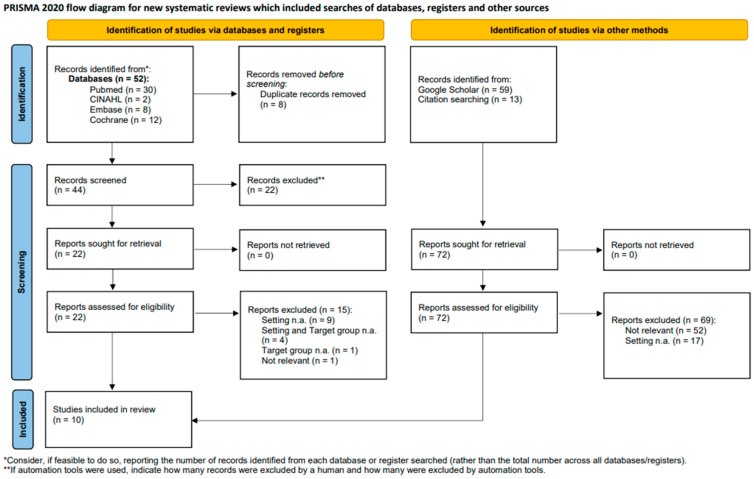
PRISMA 2020 flow diagram.

Figure 1 illustrates the research and screening process applied. A total of 10 studies met the inclusion criteria and were incorporated into the analysis.

### 3.2. Concept of a Goal-Oriented Rehabilitation Care Process in Geriatric Rehabilitation

**Figure 2:**
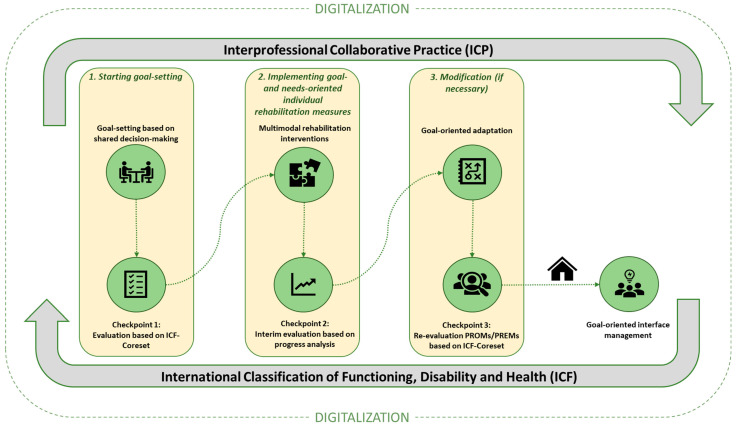
Goal-oriented geriatric rehabilitation care process.

Frailty as indicator for person-centered GR programs has proven effective and the inclusion of frailty trajectories should be considered for all older persons experiencing functional decline after hospitalization or in the community [24]. Given the multidimensional nature of frailty [25], the rehabilitation process must be tailored according to functional residual capacities, with the equivalent potential of the individual goals expressed by the patients. Figure 2 shows the integrated process care model for goal-oriented GR.

**Legend Figure 2:** Figure 2 shows the different stages of geriatric rehabilitation (GR) and how the individual preferences and believes of older patients shall be included to address person-centered and integrated goal-oriented GR. Goal-defining communication, based on the concept of shared decision making, should be included at the beginning of rehabilitation, during the care process as well as at the end of a rehabilitation care process. Any information at the end of rehabilitation should be communicated to partners along the integrated care path of older patients, such as general practitioners (GPs), community nurses, etc.

As may be seen from Figure 2, the goal-oriented care process is a multi-step and tailored care process. The baseline for any GR process is the Comprehensive Geriatric Assessment (CGA). Data from CGA inform about individual capacities on a functional level and the health of patients [8] and are transferred into the language of ICF core sets for rehabilitation. Beyond solely functional evaluation, goal discussions based on shared decision making (SDM) have been proven effective for GR outcomes in the literature [26] and allow the design of the rehabilitation process according to the needs of the patient and/or to optimize their rehabilitation, respectively [27].

Given the heterogeneous phenotype of frailty in older people, pain, depression, anxiety, loneliness, issues regarding family, the meaning of life and fear of physical or mental decline should be addressed during the goal-setting procedure. When using ICF‘s nine activity and participation areas as a common language, “individual” and “personalized” priority areas can be identified during the rehabilitation program. At the beginning of the rehabilitation, the focus is more on areas of coping with activity limitations (learning and thinking, communication, mobility and self-care) and less on limitations in participation [28]. Besides the individual and contextual factors of health in those patients, the rehabilitation setting itself seems to have an influence on how patients set their individual health goals [21]. Goals need to be personally meaningful for patients and include individual preferences and circumstances, and patients need support in attaining them [29].

Rose et al. showed that there are different levels of patient involvement in goal setting, but few teams have an entirely patient-centered approach, even though SDM can have benefits such as increased confidence, higher motivation and a sense of patient ownership [26]. The concept of SDM is not only desirable but “essential” from an ethical perspective, as goal setting involves patients in decision making and is therefore a means of meeting patients’ needs [30]. There is evidence that, at admission to rehabilitation, patients have a strong need to tell “their story” and express their individual expectation of physical, psychological and social support. Furthermore, the need for SDM in the areas of autonomy and staying independent is addressed by older people undergoing rehabilitation [31]. Well-implemented goal-setting procedures in the clinical practice of GR providers can improve the health-related quality of life, emotional well-being and emotional state (especially the self-efficacy) of patients [18].

There are different tools available to promote personalized goal setting in rehabilitation, even for older patients. The most frequently used tools, which are based on information and communication technology, are the “Canadian Occupational Performance Measure”, the “Goal setting and Action Planning”, the “Aid for Decision-making in Occupational Choice in English” and “Talking mats” [32]. According to authors’ conclusion, it seems important to notice that those goal-setting tools based on communication technologies “appeared to primarily be designed for an in-person context and not specifically for remote use” [32]. Still, the majority of the published tools for goal setting seem to focus on the level of activity and participation and involve SDM at a client level.

As there are different phases of rehabilitation, from early mobilization to classic rehabilitation treatment to follow-up care, there is strong evidence that individual rehabilitation goals fluctuate during the rehab process [33]. During early inpatient rehabilitation, the goals are related to going home, as opposed to following discharge from rehabilitation, when patients formulate new, more ambitious goals related to their premorbid level [34]. This merely explains why goal setting based on the model of SDM should be an integral part of the rehabilitation process and beyond (see Figure 2).

### 3.3. The Participatory Rehabilitation Approach in Geriatric Rehabilitation

Not only the setting of the goals but also the process of achieving them is an essential part of participatory rehabilitation [8,29]. When patients are able to participate in their rehabilitation program, this promotes their sense of control and independence, which is in line with the principles of a person-centered rehabilitation approach [6].

The participatory rehabilitation goals transferred to the ICF and shared between patients and professionals are “the backbone” for effective teamwork in rehabilitation, as priorities in the rehabilitation program can be identified [28] (see Figure 2). Discussing the goals between professionals and patients pulls away from diseases and technological possibilities and towards “what really matters” in patients’ everyday lives. This can help professionals with the paradigm shift from a disease-oriented to a genuine patient-centered approach [35]. Considering the individual goals of patients with frailty, Yamashita et al. demonstrated that the majority of older patients in rehabilitation living with frailty see their rehabilitation goals in the area of “mobility and self-care” [36]. The focus of healthcare professionals working on rehabilitation mobility programs for patients with advanced frailty reported in the literature is in accordance to patients’ preferences [36]. In particular, the focus in rehabilitation programs for patients with advanced frailty is more towards falls prevention programs [37,38,39]. Frailty and falls are two sides of a mechanistic concept, whereby falls and fear of falling increase the risk of frailty in community-dwelling older adults. The knowledge of this association is of utmost importance in clinical practice, since it can help health professionals to understand the holistic needs of older patients in rehabilitation. The concept of frailty in its various stages and trajectories seems to impact patients’ goals and may support the participation of patients in their personalized rehabilitation program, likewise the prevention and health promotion protocols [40]. If the number of overlapping areas of frailty was low, the goals relating to community and social life prevailed [40].

It could be demonstrated that nursing staff can play an important role in goal setting in GR, as they are often closest to the patient. A recent review showed that strengthening the role of nurses can improve the goal-setting process for patients and that inter-professional collaboration and clear work procedures and material support the collaborative process of “goal-talk” and increase transparency within the rehabilitation process [29].

Overall, healthcare professionals need a supportive organizational context and skills to develop a goal-oriented and participatory approach to GR [41]. A recent European initiative developed a framework of transversal interprofessional skills in the care of older people at risk of or living with frailty to allow such interprofessional collaborative practice [42]. The framework includes domains of knowledge and skills; one domain solely focuses on capabilities in interprofessional collaborative practice (ICP) and has a strong focus on communication skills. SDM is a tool supported by evidence to support the successful rehabilitation care of older people. This approach sets the clear need for interprofessional training at the under- and postgraduate levels for healthcare professionals.

Still, rehabilitation teams often struggle to integrate a client-centered, interdisciplinary model in rehabilitation. A lack of understanding in the use and advantages of goal setting to improve client outcomes and autonomy in rehabilitation are key factors for this barrier [21]. These findings are interesting, as we found evidence for the efficacy of person-centered rehabilitation in in-patient [43] as well as community-based care settings [44].

Additionally, continuity of care, time and trust in order to build a relationship with patients and the physical environment are important factors [29] (see Figure 2).

### 3.4. What Is the Future for “Goal-Oriented and Participatory” Geriatric Rehabilitation in Digitalized Healthcare?

In daily practice, GR patients are not always involved in goal setting, the communication process is often “incomplete” and focused on functional domains only. Goal setting is not merely about establishing and negotiating goals but also about involving the patient in defining and evaluating them continuously during the rehabilitation process in a participatory setting [15].

Smit et al. postulated that patients wish to be involved in a flexible and individual goal-setting process rather than in a more one-size-fits-all approach [45]. Their results showed that both professionals as well as patients have a need for patient-centered goal-setting interventions in GR. In this study, however, both expressed significant concerns about the feasibility of a collaborative functional goal setting [45].

Still, many healthcare professionals working in rehabilitation struggle to meet the need for a participatory approach, especially in GR [46]. This explains why “true” person-centered rehabilitation care is not yet fully implemented. Although there is increasing evidence for the efficacy of person-centered care on rehabilitation outcomes such as improvements in functional performance and quality of life [47], the heterogeneity of the study designs and methodologies used to achieve person-centered rehabilitation care is high.

When comparing structured goal-setting approaches to usual-care goal setting, no advantage for a structured approach was evident in terms of health-related quality of life, emotional status, participation or activity levels [48]. However, self-efficacy was higher with a structured approach. Levack et al. furthermore emphasized the importance of formulating person-centered and personally meaningful goals in rehabilitation to improve people’s quality of life, well-being and self-efficacy [48]. A systematic review concluded that standardized goal-setting procedures are not recommended in the rehabilitation of older adults, as they do not result in better physical functioning or quality of life [30]. In order to achieve an understanding of meaningful rehabilitation goals, clinicians need to reconcile their own assessments with those of the patient, as patients have a wide range of functional, as well as psychological, rehabilitation needs that are often not included in their primary evaluation reports based on ICF coding [49].

Factors like age, place of residence and the presence of cognitive impairment should also lead to the modification of interventions according to the changing needs of patients during the rehabilitation process [8].

There is international consensus that cognitive impairment should not lead to exclusion of GR, as currently demonstrated in some Western countries. Several studies show that rehabilitation goals for patients with early-stage dementia can be achieved in terms of personal independence [7]. Dutzi et al. reported that goal setting for these patients is more feasible with a structured approach, allowing a rather holistic view of possible rehabilitation goals and needs from the patient’s perspective, which are mostly about mobility and mental well-being [49]. Doctors reported barriers when clients have cognitive impairments; however, they also stated that additional training to overcome these communication barriers may be helpful to support participatory goal setting in patients living with cognitive limitations [21]. Therefore, a predictive rehabilitation model for the assessment of patients on admission would be useful for planning a patient-specific program aimed at maximizing functional independence and thus quality of life [49].

Apart from the indicators described, the impact of future digitalization in healthcare on the concept under study is still unsolved. At this point, one cannot predict the impact of telerehabilitation and/or digital/artificial intelligence supporting the personalized rehabilitation process in the near future. A recent study showed that the use of machine learning and artificial intelligence makes it possible to make medical knowledge technically usable in a structured form in order to individually personalize activities for the patient within the framework of a virtual coaching scenario [50].

Independent from technology, however, SDM and communication will remain key elements in ensuring participatory rehabilitation, as social participation and the related goals are of particular importance for the entire rehabilitation process. In a recently published review [51], an overview of the technologies for goal setting in the rehabilitation of adults was collected. The identified technologies for use in adult rehabilitation were limited but largely accepted and valued by patients according to this scoping review. According to the cited paper, the technologies provided choices for patients, from a predetermined list of personalized rehabilitation goals to online meetings to decide on shared objectives for rehabilitation. However, many of the papers included in this review were not developed on underpinning communication theories and none was tested specifically in older patients. The goals set in the trials described were also monitored remotely during rehabilitation, primarily using pedometry as functional parameter, followed by asynchronous communication between patients and rehabilitation teams or by online face-to-face communication. Another review focused on the usability of ICT tools in remote rehabilitation and their evaluation from patients’ as well as professionals’ perspectives [52]. Most SDM technologies described were web based, and the authors detected 14 different methods to evaluate the usability of the tools. The most frequently used tools for evaluation in ICT-based SDM in rehabilitation were questionnaires and semi-structured interviews. User-centered design was the most frequently used technology design framework. The results from this review highlighted the complexity of technology use in SDM and the large variety of usability evaluation frameworks applied in the literature. The predominant evaluation domain selected in the studies included was the “satisfaction” of patients and carers [52]. This is an important and promising finding for the near future in GR, as SDM is often a struggle due to patients frequently being hesitant or not interested in promoting their own ideas in goal setting, as they rely on health professionals to determine their rehabilitation planning. Healthcare professionals, in turn, are often overwhelmed by the task of collaborating or not trained in goal setting, even in a non-digitalized environment [53,54].

## 4. Conclusions and Future Directions

Personalized goal setting and a participatory rehabilitation approach are “key indicators” of sustainable GR. Vulnerable people and those living with frailty especially, need to be included in the goal setting and participatory rehabilitation processes. CGA and SDM during the goal-setting process allow a comprehensive care approach separate from solely function-based rehabilitation programs. To aim for such comprehensive and participatory care programs, the professionals involved need to be equipped with knowledge, skills and attitudes to run these programs favorably and on an interdisciplinary basis. This innovative concept for GR requires commitment from care providers and a structural framework, which supports inter-professional involvement in the rehabilitation care process.

Using goals as well as practical advice can improve patient-centered working in the context of goals for both patients and professionals. In our opinion, only the interaction of interdisciplinary professional groups, the alignment of medical, therapeutic and nursing activities, the involvement of the patient in the rehabilitation process and individualized discharge management will sustainably improve the outcome for geriatric patients.

In the future, the use of artificial intelligence (AI) and telerehabilitation will also be an important topic for geriatric patients. However, precise inclusion criteria should be defined in order to clarify for which populations of multimorbid, frail patients at risk of falling that telerehabilitation is suitable. It seems noteworthy that it is more difficult to set rehabilitation goals in this setting than in face-to-face contact in a rehabilitation facility. However, the use of machine learning and artificial intelligence can make it possible to make medical knowledge technically usable in a structured form in order to individually personalize activities for the patient.

Further research to develop a shared understanding of evidence-based goal setting and participatory rehabilitation among experts and clinicians, as well as standardized guideline protocols for the GR of people living with frailty (also in digital healthcare) and economic protocols to examine the return on investment in integrated care models is needed. Developing standards, especially in the light of a rapidly developing digitalized healthcare environment, will support rehabilitation approaches towards a more effective and individualized care for our older patients.
